# Genomic and physiological characterization of newly isolated nitrous oxide-reducing bacterium *Stutzerimonas frequens* strain E49 from landfill leachate-treating activated sludge

**DOI:** 10.1128/aem.00743-26

**Published:** 2026-08-31

**Authors:** Jameil Magomnang, Shohei Yasuda, Akihiko Terada

**Affiliations:** 1 Department of Applied Physics and Chemical Engineering, Tokyo University of Agriculture and Technology 13125, Koganei, Tokyo, Japan; 2 Civil Engineering, School of Engineering, College of Science and Engineering, University of Galway 8799, Galway, Ireland; 3 Global Innovation Research Institute, Tokyo University of Agriculture and Technology 13125, Fuchu, Tokyo, Japan; University of Minnesota Twin Cities, St. Paul, Minnesota, USA

**Keywords:** denitrification, nitrous oxide (N_2_O) reduction, N_2_O-reducing bacteria, wastewater treatment, genome sequencing, *nosZ*, ectoine

## Abstract

Nitrous oxide (N_2_O) is a potent greenhouse gas and ozone-depleting substance, with a global warming potential 273 times greater than CO_2_ over a 100-year horizon. Microbial reduction of N_2_O to dinitrogen represents a key pathway for mitigating emissions under diverse environmental conditions. Here, we report the first comprehensive genomic and physiological characterization of *Stutzerimonas frequens* strain E49, a newly isolated N_2_O-reducing bacterium obtained from activated sludge treating landfill leachate. Whole-genome sequencing revealed a 4.51-Mbp circular chromosome and a 35.3-kbp plasmid with high completeness. Functional annotation identified a complete denitrification gene set, including *nosZ*, as well as the *ectABCD*-ask gene cluster associated with ectoine biosynthesis, suggesting adaptation to osmotic stress. Strain E49, a uniform rod-shaped bacterium (1.5–2.5 μm), efficiently reduced N_2_O under anaerobic conditions in the absence of an externally supplied organic carbon source, achieving a biomass-specific rate of 0.70 ± 0.02 µmol-N_2_O/mg-biomass/h and a cell-specific rate of 7.93 ± 0.23 × 10^−10^ µmol-N_2_O/cell/h. Among the cultivation regimens tested, nitrate-free DSMZ 1180 medium supplemented with NH_4_Cl yielded the highest activity, indicating medium-dependent regulation of N_2_O respiration. Comparative analysis showed that strain E49 outperformed several reported N_2_O-reducing isolates under carbon-limited conditions. These findings demonstrate the metabolic versatility of strain E49 and highlight its potential role as a biological sink for N_2_O in low-nutrient environments.

**IMPORTANCE:**

Nitrous oxide is a powerful greenhouse gas that contributes to climate change and ozone depletion. Microorganisms that convert nitrous oxide into nitrogen gas play an essential role in reducing these emissions. In this study, we investigated *Stutzerimonas frequens* strain E49, a bacterium isolated from wastewater treatment sludge. We found that this organism can efficiently reduce nitrous oxide even without an external supply of organic carbon, which is typically required by most bacteria. This suggests that the bacterium can rely on internal energy reserves to carry out this process. We also identified the genetic basis for its nitrous oxide reduction and its ability to adapt to environmental stress. These findings improve our understanding of how nitrous oxide-reducing bacteria function in nutrient-limited environments and may support the development of strategies to mitigate emissions in wastewater treatment and other engineered systems.

## INTRODUCTION

Nitrous oxide (N_2_O) is a potent greenhouse gas with a global warming potential approximately 273 times that of carbon dioxide over a 100-year timescale, contributing significantly to climate change and stratospheric ozone depletion (1, 2). Anthropogenic N_2_O emissions largely arise from agricultural practices and wastewater treatment, where microbial nitrogen transformations play central roles. Consequently, mitigating N_2_O emissions has become a critical environmental challenge, particularly in engineered microbial systems, such as wastewater treatment plants (WWTPs) (3).

Denitrification, the sequential enzymatic reduction of nitrate to dinitrogen gas under oxygen-limited conditions, is a major contributor to N_2_O emissions when the process is incomplete (4). In many wastewater treatment systems, suboptimal conditions such as fluctuating oxygen levels, carbon limitation, or high salinity can hinder complete denitrification, leading to the accumulation of N_2_O as an intermediate. Certain microorganisms possess the *nosZ* gene, which encodes nitrous oxide reductase (NosZ), the enzyme that catalyzes the final step of denitrification, converting N_2_O to inert N_2_ (5). Identifying and understanding these N_2_O-reducing bacteria (N_2_ORB) is therefore essential for developing microbial strategies to mitigate N_2_O emissions in both natural and engineered environments.

Among potential high-risk sites for N_2_O emissions, landfill leachate treatment systems are particularly important (6). Leachates often contain high concentrations of nitrogen compounds and salts, creating extreme and copiotrophic conditions for microorganisms (7). High salinity, nearly half that of seawater in some cases, poses additional osmotic stress, which may influence denitrification efficiency and N_2_O emission dynamics (8–10). Understanding the ecophysiology of N_2_ORB in such environments is crucial for both emission mitigation and potential resource recovery.

The genus *Stutzerimonas*, recently reclassified from the *Pseudomonas* phylogenetic group, comprises metabolically diverse denitrifying bacteria within the *Pseudomonadaceae* family (11). Some species harbor the complete denitrification pathway and exhibit excellent N_2_O-reduction performance (12–16). Exploration of their diverse habitats, including stressful environments, expanded their phylogenies, leading to the discovery of novel species, such as *Stutzerimonas frequens* and *Stutzerimonas degradans* (17). Despite their genomic and ecological potential, there is limited information on their genome content, N_2_O-reduction physiology, and tolerance to high-salinity or oligotrophic conditions.

In this study, we aimed to isolate and characterize N_2_O-reducing *Stutzerimonas* from high-salinity landfill leachate. Given that the successful isolation of N_2_ORB depends heavily on the enrichment of targeted N_2_O reducers, we employed a membrane biofilm reactor (MBfR, Fig. S1), which enables selective enrichment of high-affinity N_2_O-reducing bacteria under controlled anaerobic conditions. We report the isolation of *S. frequens* strain E49 and its complete genome sequencing, which revealed the presence of a full denitrification pathway, including the *nosZ* operon, and the *ectABCD-ask* gene cluster, responsible for the biosynthesis of the compatible solutes ectoine and hydroxyectoine, which confer osmoprotection in saline environments. We further characterized the strain’s N_2_O-reduction activity under anaerobic conditions, in the absence of external organic carbon, to assess its intrinsic respiratory capacity independent of external electron donors, thereby reflecting potential performance in oligotrophic environments, where carbon limitation may occur. Additionally, we evaluated the influence of prior cultivation history on respiratory performance, since physiological traits relevant for environmental applications can be affected by growth conditions (18–20).

Overall, this study provides novel insights into the genomic and physiological traits of a newly isolated N_2_O-reducing bacterium from high-salinity wastewater. These findings highlight the potential of *S. frequens* E49 to mitigate N_2_O emissions and support sustainable resource recovery applications through its osmotolerance and metabolic versatility.

## RESULTS AND DISCUSSION

### Isolation and genome analysis

A single bacterial strain was successfully isolated from activated sludge through three successive rounds of limiting dilution. Genomic DNA was extracted from the pure culture and sequenced using a hybrid approach combining long- and short-read data. Following quality filtering and adapter trimming, a total of 114,857 long reads (759.8 Mbp) and 16.67 million short reads (2.42 Gbp) were obtained.

**Fig 1:**
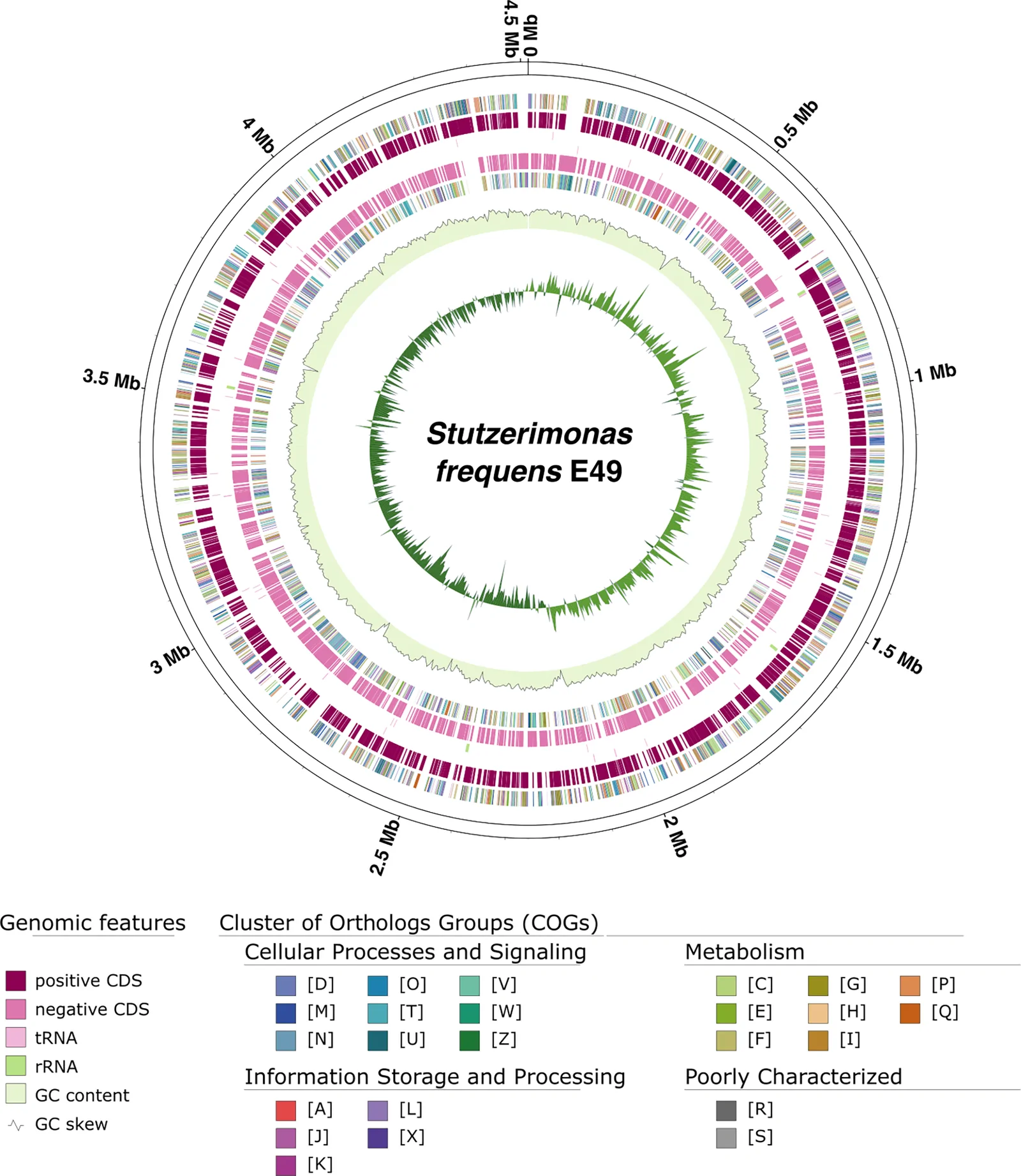
Complete genome structure of *S. frequens* strain E49, isolated from activated sludge after three rounds of limiting dilution. Genome assembly yielded a circular chromosome (4,514,130 bp) and a circular plasmid (35,333 bp). The genome has a GC content of 63.6%, with 4,208 CDSs, a coding density of 89.8%, 12 rRNA genes, and 65 tRNA genes. No gaps were present, and genome completeness was confirmed at 100%. The circular map displays forward- and reverse-strand CDSs, rRNA/tRNA loci, and GC content/skew across the genome.

Genome assembly was performed using polished long reads, followed by correction with short-read data. This yielded a complete, gapless genome consisting of a circular chromosome (4,514,130 bp) and a circular plasmid (35,333 bp) (Fig. 1). The GC content was 63.6%, with 4,208 predicted coding sequences (CDSs), a high coding density (89.8%), and 100% completeness. A total of 12 rRNA and 65 tRNA genes were identified.

**Fig 2:**
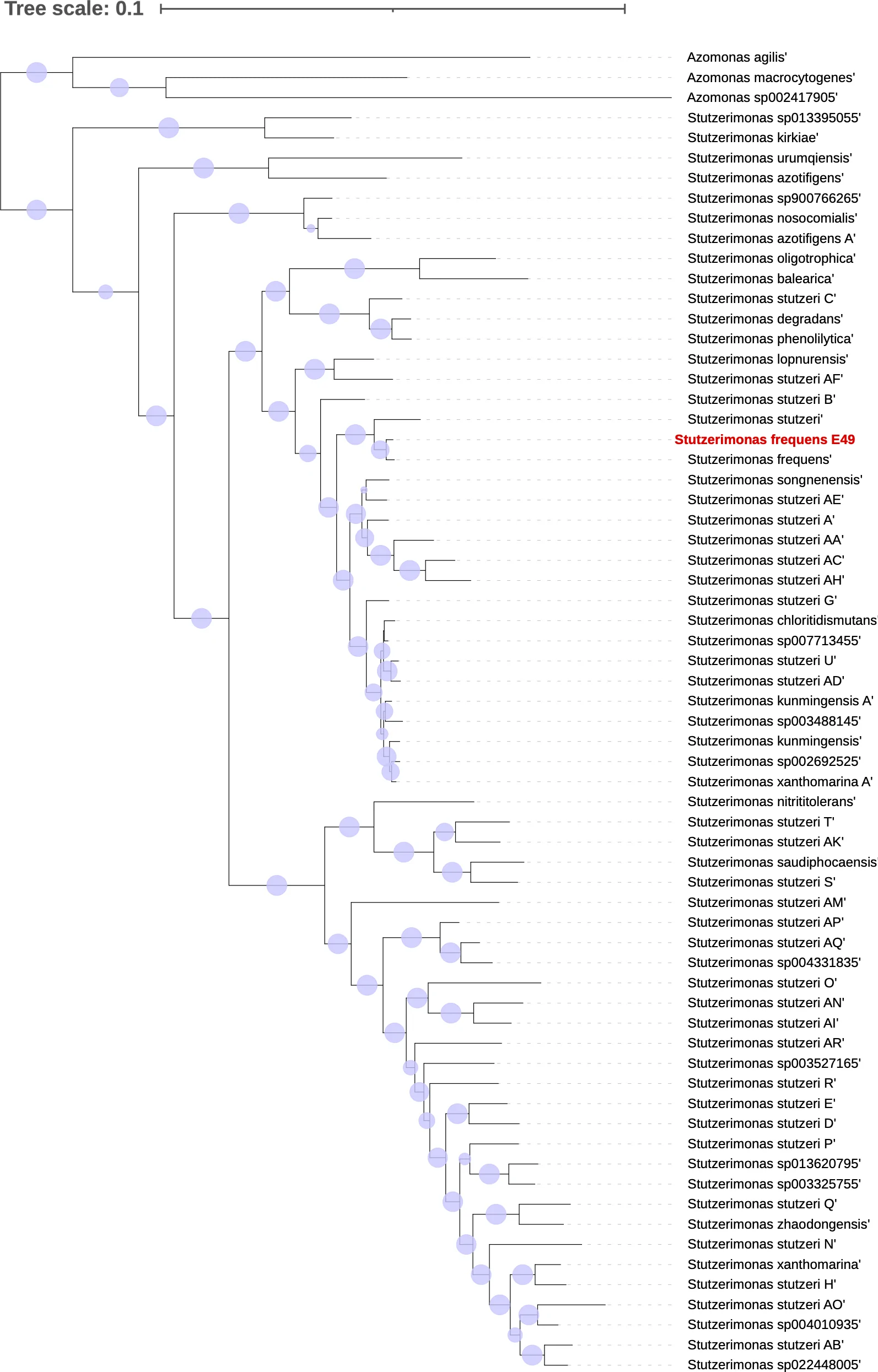
Phylogenetic classification of *S. frequens* strain E49 based on whole-genome analysis. Phylogenetic reconstruction using GTDB-Tk (de_novo_wf with 120 marker genes) classified the isolate within the genus *Stutzerimonas*, a member of the class *Gammaproteobacteria*. Strain E49 showed the highest similarity to *S. frequens* (RefSeq: GCF_024448335), with an average nucleotide identity (ANI) of 97.11%, exceeding the 95% species delineation threshold. Accordingly, the isolate was assigned to the same species and designated *S. frequens* E49.

Phylogenomic analysis based on average nucleotide identity (ANI) classified the isolate within the genus *Stutzerimonas* (formerly *Pseudomonas*), belonging to the class *Gammaproteobacteria*. The strain showed 97.11% ANI similarity to *S. frequens* (RefSeq: GCF_024448335), exceeding the 95% species threshold, thereby confirming its species-level identity (Fig. 2). The isolate was designated *S. frequens* strain E49.

### Functional gene annotation of strain E49

The translated amino acid sequences of strain E49 were mapped to the Kyoto Encyclopedia of Genes and Genomes (KEGG) reference pathways to assess their genetic potential. The genome harbors a complete denitrification gene set, including nitrate reductase gene (*narGHI*), nitrite reductase gene (*nirS*), nitric oxide reductase gene (*norBC*), and clade I nitrous oxide reductase gene (*nosZ*) (Fig. S2), indicating its capacity to perform complete denitrification to dinitrogen gas.

Strain E49 lacks key genes involved in methane and methanol oxidation pathways, including the *pmoABC* operon encoding particulate methane monooxygenase (pMMO, Fig. S3), as well as *mxaACDFGIJKL* and *xoxF*, which encode methanol dehydrogenases. Furthermore, preliminary anaerobic assays showed no detectable CH_4_ consumption (Fig. S4). Collectively, these genomic and physiological findings indicate that strain E49 is not a methylotroph, does not rely on C1 compounds for carbon or energy metabolism, and is unlikely to possess anaerobic methane oxidation capability under the conditions examined in this study.

Additionally, the genome encodes the ectoine biosynthesis cluster (*ectABCD-ask*), directing the production of ectoine and hydroxyectoine, compatible solutes that confer osmotic stress protection, with hydroxyectoine providing enhanced stabilization of proteins and cellular membranes under extreme conditions (Fig. S5). Within this cluster, *ectA*, *ectB*, and *ectC* encode the enzymes for ectoine synthesis—N-*γ*-acetyltransferase, L-2,4-diaminobutyric acid transaminase, and ectoine synthase, respectively—while *ectD* encodes ectoine hydroxylase for hydroxylation to hydroxyectoine, and *ask* encodes aspartate kinase, which provides the key precursor, aspartate *β*-semialdehyde, enhancing flux toward ectoine biosynthesis. Strain E49 harbors this cluster as an adaptive mechanism to mitigate osmotic stress: the coordinated synthesis of ectoine and hydroxyectoine stabilizes proteins and cellular membranes under high-salinity or nutrient-limited conditions. Notably, genes involved in ectoine degradation (*doeA* and *doeB*) are absent, thereby facilitating intracellular accumulation and maintaining a sustained reservoir of compatible solutes for cellular homeostasis.

In comparison, other species historically classified as *Pseudomonas*—including several strains now reclassified as *Stutzerimonas*—typically harbor only orphan *ectC* genes (21, 22), whereas the complete cluster is largely conserved in *Pseudomonas stutzeri* strains (23–25). Notably, the successful isolation of strain E49 from an MBfR operated under high-saline conditions (3 wt% NaCl) was likely achieved through enrichment for halotolerant or osmoadapted microorganisms, thereby favoring the growth of this novel isolate. To our knowledge, strain E49 represents the first *S. frequens* isolate reported to possess a complete ectoine biosynthesis cluster, indicating a strain- and species-specific genomic feature. From both ecological and biotechnological perspectives, the presence of *ask* and the complete *ectABCD* cluster, together with the absence of ectoine degradation genes, supports the intracellular accumulation of compatible solutes, enhancing osmotic stress tolerance under fluctuating or oligotrophic environmental conditions and suggesting potential for sustainable resource recovery through ectoine and hydroxyectoine production (26, 27).

### Visualization and morphological characterization of strain E49

**Fig 3:**
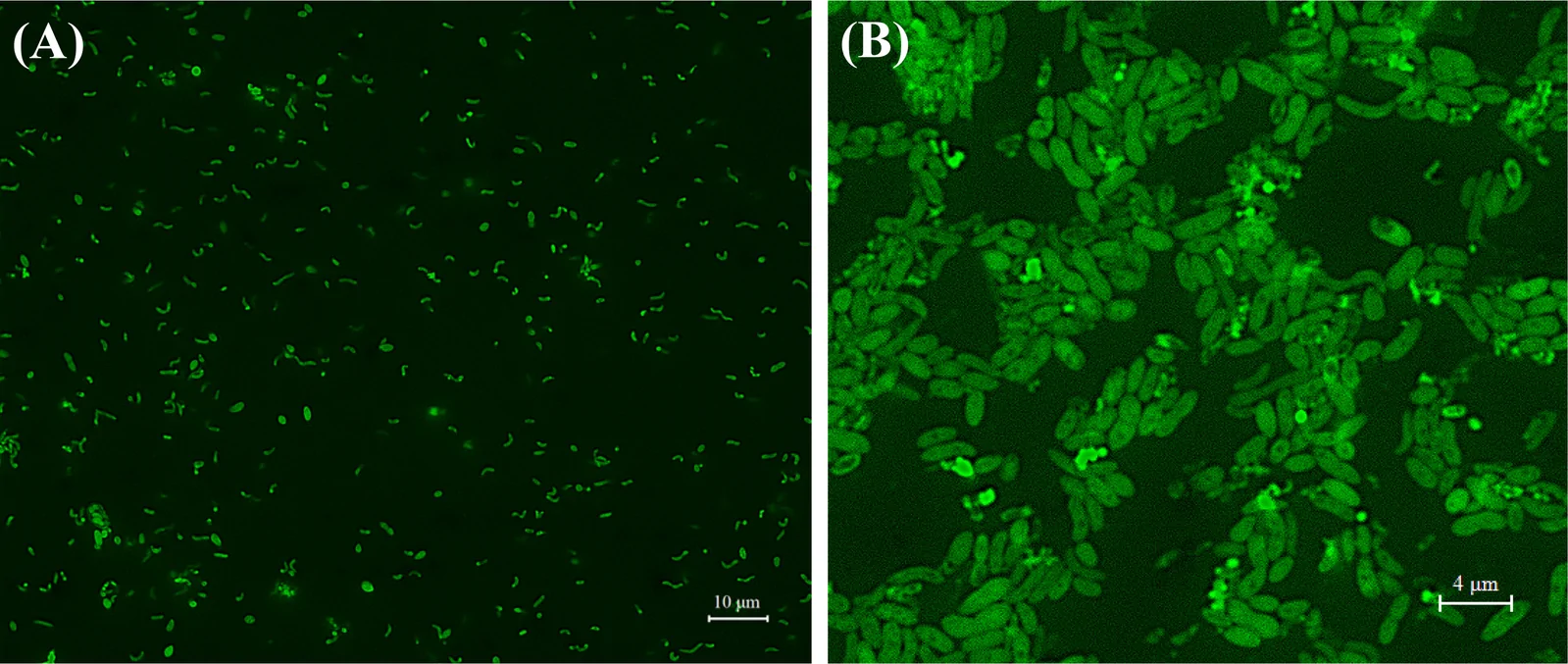
*S. frequens* strain E49 cells stained with SYBR Green I and visualized using a BZ-X800 all-in-one fluorescence microscope equipped with a filter set (excitation: 488 nm; emission: 520 nm). Panel **A** shows fluorescence-based cell enumeration under 100× oil immersion. Panel **B** highlights the cell morphology, revealing the typical straight-to-slightly curved rod-shaped structure.

The population overview (Fig. 3A) revealed even cell distribution, consistent fluorescence intensity, and the absence of debris or morphological contaminants, confirming culture uniformity. High-magnification images (Fig. 3B) showed cells with straight-to-slightly curved rod-shaped structures measuring 1.5–2.5 μm, consistent with previously described *Stutzerimonas* species (28, 29). These observations demonstrate the uniformity and typical morphology of *S. frequens* strain E49.

Cells were frequently observed in small aggregates, potentially indicative of active growth and early-stage aggregation—a behavior consistent with initial biofilm development in *Pseudomonadaceae* (30, 31). However, extracellular polymeric substances (EPS) or biofilm matrix components were not specifically assessed, and while dedicated EPS-specific staining or biofilm assays were not performed, such methods could be applied in future work to confirm nascent biofilm formation.

### Medium-dependent regulation of N_2_O reduction

**Fig 4:**
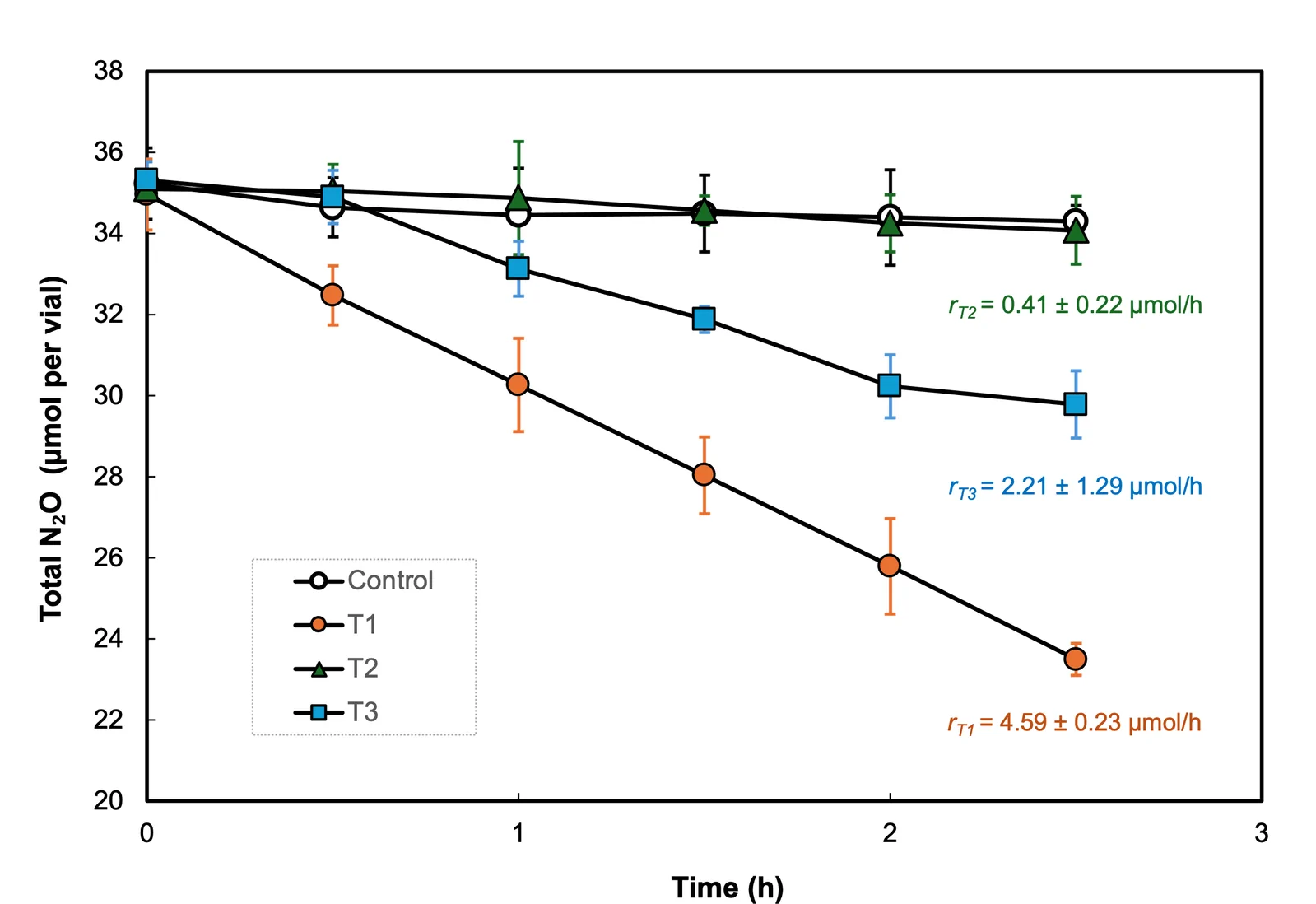
Time course of N_2_O consumption by *S. frequens* strain E49 under anaerobic batch incubation. Total N_2_O concentrations (headspace + dissolved; µmol) were measured over a 2.5-h period for three cultivation regimens and a non-inoculated control. The regimens were as follows: (T1) pre-culture and activity test in DSMZ 1180 medium, (T2) pre-culture in JCM 12 and activity test in DSMZ 1180, and (T3) pre-culture and activity test in JCM 12. Data represent mean values from biological triplicates (*n* = 3), with error bars indicating standard deviation (*n* = 3).

Strain E49 exhibited pronounced N_2_O-reduction activity under different cultivation regimens (please see the experimental design in Fig. S6). The highest N_2_O consumption rate was observed under the T1 condition (4.59 ± 0.23 µmol-N_2_O/h; 0.71 ± 0.04 µmol-N_2_O/mg-biomass/h), followed by T2 (2.21 ± 1.29 µmol-N_2_O/h; 0.34 ± 0.20 µmol-N_2_O/mg-biomass/h), and T3 (0.41 ± 0.22 µmol-N_2_O/h; 0.063 ± 0.034 µmol-N_2_O/mg-biomass/h) (Fig. 4). These results demonstrate that both medium composition and consistency between pre-culture and assay conditions strongly influence the physiological state supporting N_2_O respiration. Relative to T1, the N_2_O reduction rate decreased by approximately 52% under T2 and 91% under T3, highlighting the substantial impact of cultivation history and medium composition on respiratory performance. The intermediate activity observed in T2 suggests that pre-culture conditions alone can influence subsequent N_2_O reduction, whereas the markedly lower activity in T3 indicates that combined differences in both pre-culture and assay media further suppress respiratory capacity.

The superior performance observed in T1, conducted in nitrate-free, ammonium-enriched modified DSMZ 1180 medium without added organic carbon, indicated that strain E49 efficiently activated the *nosZ*-encoded N_2_O reductase system under carbon-limited conditions. This is consistent with previous observations in other N_2_O-reducing bacteria (18, 32, 33).

As a follow-up, the total organic carbon (TOC) analysis confirmed that residual organic carbon in the assay system was negligible (~0.012 mg/L), supporting that N_2_O reduction was not driven by external or carryover carbon sources. Thus, the observed activity reflects intrinsic respiratory capacity rather than heterotrophic growth-linked metabolism (Table S1). However, the intracellular reserves supporting N_2_O reduction are likely finite, and sustained activity over extended periods would presumably require replenishment of electron donors. Therefore, while strain E49 demonstrated N_2_O reduction in the absence of externally supplied carbon during the assay period, the duration for which this activity can be maintained remains unknown and warrants further investigation.

The notably low activity observed in T3 (JCM 12 medium) suggests that pre-cultivation in a complex organic medium may shift metabolic allocation toward growth-related pathways or delay the activation of the N_2_O respiratory system. Because JCM 12 contains complex organic substrates (peptone and meat extract), the cells may preferentially allocate resources toward biomass production and general heterotrophic metabolism rather than maintaining physiological readiness for N_2_O respiration. In contrast, DSMZ 1180 is a defined mineral medium containing inorganic carbon (carbonate) but no added organic carbon, more closely resembling the enrichment conditions used during isolation of strain E49 and therefore favoring physiological adaptation to N_2_O reduction.

In addition to differences in carbon source composition, salinity may also have contributed to the observed responses. DSMZ 1180 contains 30 g/L NaCl, whereas JCM 12 contains 5 g/L NaCl. Given that strain E49 was enriched from an MBfR (Fig. S1) operated at 3 wt% NaCl and harbors a complete *ectABCD-ask* cluster involved in ectoine and hydroxyectoine biosynthesis, elevated salinity may promote osmoadaptive responses that influence subsequent respiratory activity. However, because salinity and carbon source composition varied simultaneously in the present study, their individual contributions cannot be distinguished.

While these results show that inorganic, ammonium-enriched conditions can support N_2_O reduction, additional cultivation experiments, including replicates under JCM 12 conditions, would strengthen these conclusions. Future studies employing factorial manipulation of salinity and carbon source composition would help clarify their respective roles in regulating N_2_O reduction by strain E49. Overall, these findings demonstrate that inorganic, ammonium-enriched, nitrate-free conditions promote strong and consistent N_2_O reduction in strain E49. Therefore, the T1 regimen was chosen for the subsequent vial experiments to ensure maximum and physiologically stable respiratory activity.

### N_2_O reduction performance of strain E49

After 2.5-h incubation (OD_600_ = 2.83 ± 0.07), strain E49, which was acclimatized and tested by DSMZ 1180, consumed 11.5 ± 0.8 µmol-N_2_O, corresponding to an average consumption rate of 4.59 ± 0.13 µmol-N_2_O/h. This equated to a biomass-specific reduction rate of 0.70 ± 0.02 µmol-N_2_O/mg-biomass/h and a cell-specific rate of 7.93 ± 0.23 × 10^−10^ µmol-N_2_O/cell/h (Table 1). Strain E49’s performance under carbon-limited conditions compares favorably with known N_2_O reducers. Its biomass-specific rates fall within the lower-to-moderate range observed for pure cultures of N_2_O reducers, such as *Azospira* sp. I13 (6.13 µmol-N_2_O/mg-biomass/h) and *Shewanella loihica* PV-4 (26.8 ± 1.4 µmol-N_2_O/mg-biomass/h), and are of the same order of magnitude as rates reported for activated sludge communities (1.16 ± 0.06 µmol-N_2_O/mg-biomass/h) (13, 34, 35). Furthermore, it is comparable to rates from *Pseudomonas* isolates, which range from 2.2 to 2.6 µmol-N_2_O/mg-biomass/h (36). By contrast, Yoon et al. (13) reported a notably higher rate of 249.6 µmol-N_2_O/mg-biomass/h, highlighting that N_2_O reduction capacity can vary widely depending on the strain, experimental conditions, and biomass estimation methods. Notably, the rates reported here reflect the specific conditions tested, rather than a maximal rate, which may account for its lower value relative to some previously reported pure cultures.

**TABLE 1:** Biomass and cell-specific N_2_O reduction rates of *S. frequens* strain E49^*a*^

Sample	Biomass wt. [mg]	Cell conc. [×10^8^ cell/mL]	*r*_avg_ [µmol-N_2_O/h]	*v*_biomass_ [µmol-N_2_O/mg-biomass/h]	*v*_cell_ [×10^−10^ µmol-N_2_O/cell/h]
A	6.4	1.76	4.53 ± 0.12	0.71 ± 0.02	8.00 ± 0.21
B	6.7	1.83	4.64 ± 0.15	0.69 ± 0.02	7.87 ± 0.26
Average	–	–	4.59 ± 0.13	0.70 ± 0.02	7.93 ± 0.23

^*a*^ Samples A and B represent replicate vials containing cultures of *S. frequens* strain E49. Data are presented as mean ± standard deviation (SD) from triplicate measurements (*n* = 3). Biomass wt. = dry cell weight (DCW) (mg), cell conc. = cell concentration (cell/mL), *r* = N_2_O consumption rate (µmol-N_2_O/h), and *v*_biomass_ = biomass-specific N_2_O reduction rate (µmol-N_2_O/mg-biomass/h). Biomass weight is based on dry cell weight. *v*_cell_ = cell-specific N_2_O reduction rate (µmol-N_2_O/cell/h). A dash (–) indicates not applicable, as biomass weight and cell concentration were determined separately for each replicate and are therefore not presented as average values.

Among clade I *nosZ* denitrifiers, *Paracoccus denitrificans* NBRC102528 exhibits lower cell-specific rates ranging from 5.01 ± 1.00 × 10^−10^ to 5.10 ± 0.40 × 10^−10^ µmol-N_2_O/cell/h under acetate supplementation, with decreased activity when alternative carbon sources are provided (34, 37). By contrast, strain E49 achieved a higher specific activity without any added carbon source, surpassing this well-characterized clade I *nosZ* denitrifier under more stringent, carbon-limited conditions.

Interestingly, strain E49 demonstrated higher N_2_O reduction rates in the absence of externally supplied organic carbon than several clade II *nosZ* microorganisms, which are traditionally associated with higher N_2_O affinity and oligotrophic tolerance (13, 38). This suggests that strain E49 may possess distinct regulatory or enzymatic adaptations enabling elevated *nosZ* activity even in the absence of organic carbon. Further investigations are warranted to elucidate the molecular basis of this adaptation.

### Effect of acetate supplementation on N_2_O reduction

**Fig 5:**
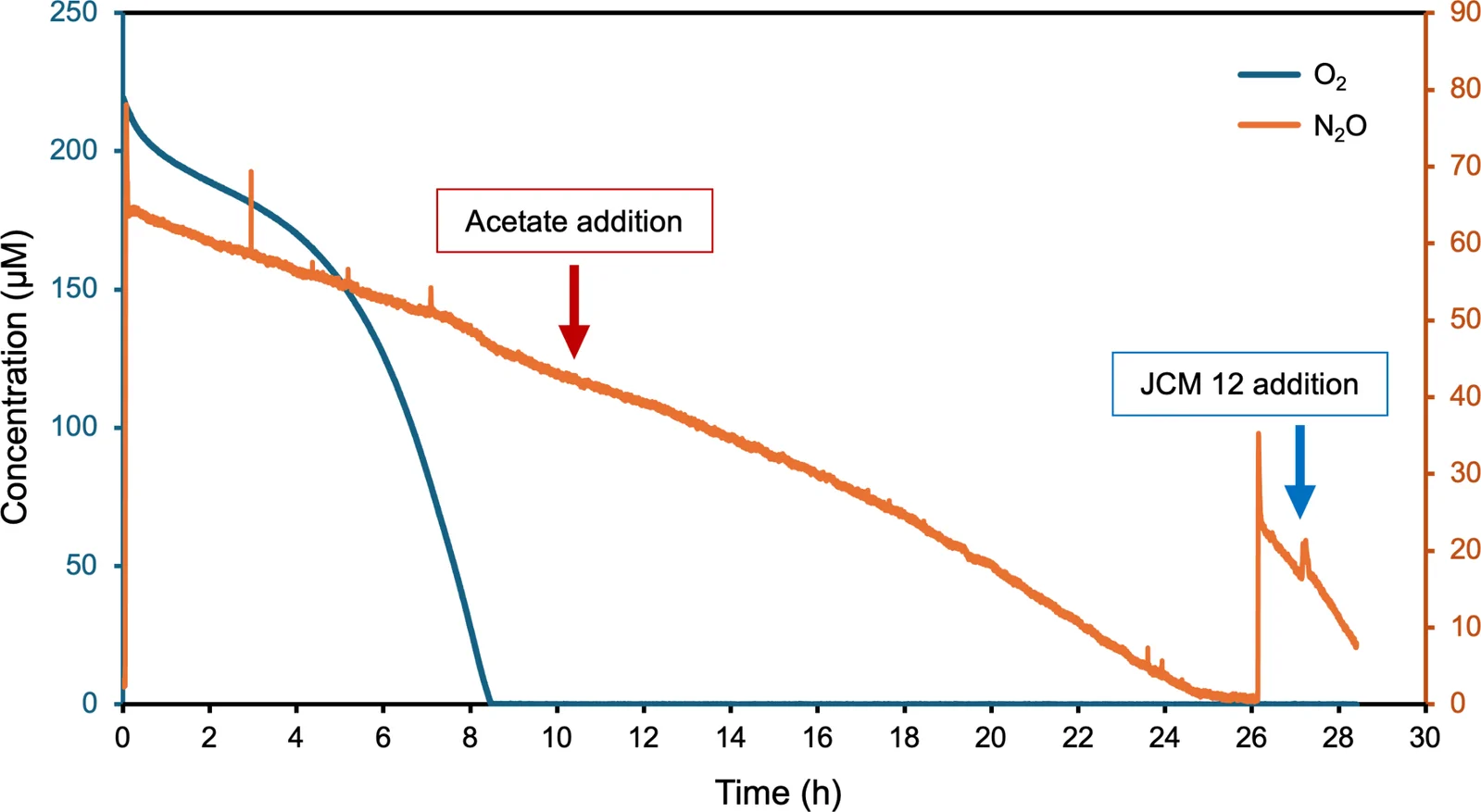
Microrespirometric profiles of *S. frequens* strain E49 during N_2_O reduction with acetate supplementation. Dissolved oxygen (O_2_, blue line) and N_2_O (orange line) concentrations were continuously monitored over time. Acetate was added after oxygen depletion to evaluate whether an externally supplied organic carbon source stimulates N_2_O reduction. No appreciable change in the N_2_O reduction profile was observed following acetate addition. After the initial N_2_O was nearly depleted, JCM 12 medium and a second dose of N_2_O were added as a positive control to confirm that the cells remained metabolically active and capable of resuming respiratory activity.

To investigate whether an externally supplied organic carbon source could stimulate N_2_O reduction by *S. frequens* strain E49, a microrespirometric assay was performed using acetate as a representative substrate (Fig. 5). Acetate was selected because it is a well-established electron donor for many heterotrophic N_2_O-reducing bacteria (18, 37), and genomic analysis indicated that strain E49 possesses the metabolic potential for acetate utilization (Fig. S7). Dissolved oxygen (DO) and N_2_O concentrations were continuously monitored before and after acetate supplementation to evaluate the respiratory response.

Following DO depletion, acetate supplementation did not produce an appreciable change in the N_2_O reduction profile (Fig. 5), indicating that the addition of an external organic carbon source in the form of acetate did not stimulate N_2_O reduction under the conditions tested. After N_2_O was nearly depleted, JCM 12 medium and a second dose of N_2_O were added, immediately restoring the respiratory activity. This confirmed that the cells remained metabolically active throughout the experiment and that the absence of an acetate-induced response was not attributable to loss of cell viability.

To validate the microrespirometric assay, *Azospira* sp. I13, a well-characterized heterotrophic N_2_O-reducing bacterium (34), was analyzed under comparable conditions. In contrast to strain E49, *Azospira* sp. I13 exhibited a clear respiratory response following acetate supplementation (Fig. S8), demonstrating that the assay was capable of detecting respiratory responses to acetate.

Collectively, these findings indicate that acetate supplementation did not stimulate N_2_O reduction by strain E49 despite maintaining cellular respiratory activity, suggesting that acetate is unlikely to serve as the primary external electron donor supporting N_2_O reduction under the experimental conditions.

### Potential electron donor for N_2_O reduction by strain E49

An electron donor is required to support N_2_O reduction by strain E49. The microrespirometric assay demonstrated that acetate supplementation did not stimulate N_2_O reduction (Fig. 5). To further investigate this observation, KEGG pathway analysis was performed. The acetate metabolism pathway was largely conserved in strain E49 (Fig. S7), including the acetate transporter ActP and the key enzymes phosphate acetyltransferase (*pta*; EC 2.3.1.8), acetate kinase (*ackA*; EC 2.7.2.1), and succinyl-CoA:acetate CoA-transferase (*aarC/cat1*; EC 2.8.3.18), which collectively facilitate acetate uptake, activation, and assimilation into central carbon metabolism. These genomic features indicate that strain E49 possesses the metabolic capacity to assimilate acetate. Nevertheless, acetate supplementation failed to enhance N_2_O reduction, suggesting that acetate catabolism was not the limiting factor governing N_2_O respiration under the tested conditions. Instead, the observed respiratory activity was likely sustained by endogenous reducing power accumulated during pre-cultivation or by an alternative metabolic pathway that remains unidentified.

To investigate alternative electron donor pathways that could sustain N_2_O reduction in the absence of acetate stimulation, additional KEGG pathway analyses were performed. Strain E49 harbors genes for assimilatory sulfate reduction (cys pathway) for the biosynthesis of sulfur-containing amino acids but lacks the complete oxidative sulfur pathway for energy generation, indicating that it cannot perform the entire chemolithotrophic sulfur oxidation (Fig. S9). There is no dedicated hydrogen (H_2_) oxidation pathway for strain E49. While some hydrogenase genes exist in KEGG as individual KO entries, they are distributed across different metabolic contexts and are not consistently integrated into a defined hydrogen metabolism pathway. BlastKOALA annotation (https://www.kegg.jp/blastkoala/) further confirmed that strain E49 lacks the canonical hydrogenase genes required for H_2_ oxidation and those required for chemolithotrophic ferrous iron (Fe²^+^) oxidation. Specifically, rusticyanin and iron:rusticyanin reductase (K20150), which are essential for electron transfer from Fe²^+^ to the respiratory chain, were not detected. Taken together, the physiological evidence and genomic features suggest that strain E49 is unlikely to rely on either externally supplied acetate or known autotrophic electron donors to sustain N_2_O reduction under the tested conditions.

Another potential mechanism is likely the supply of reducing power from intracellularly stored compounds. The preincubation with organic carbon may allow E49 to accumulate intracellular storage compounds, which could subsequently be utilized when N_2_O was supplied without external carbon addition or other readily available electron donors. The genome of *S. frequens* strain E49 encodes both polyphosphate kinase 1 (PPK1; EC 2.7.4.1) and exopolyphosphatase (PPX; EC 3.6.1.11), as highlighted in Figs. S10 and S11, respectively. These enzymes constitute a functional polyphosphate cycle, enabling reversible synthesis and degradation of polyphosphate. This system can serve as an alternative energy reserve, generating ATP through polyphosphate hydrolysis when organic carbon sources are limited. Therefore, polyphosphate metabolism may contribute to cellular energy maintenance during N_2_O reduction in the absence of externally supplied carbon. However, the direct source of reducing equivalents required for N_2_O reduction remains unclear, and the specific role of polyphosphate metabolism in supporting N_2_O respiration has yet to be experimentally verified.

The glycogen biosynthesis pathway in strain E49 is incomplete (Fig. S12). While *glgA* and *glgB* are present, the strain lacks *glgC*, which is essential for producing ADP-glucose and for *de novo* glycogen synthesis (39). Consequently, glycogen cannot serve as a direct energy or carbon source to support N_2_O reduction under carbon-limited conditions, although it may provide reducing power for other processes, such as polyphosphate synthesis. KEGG mapping further indicates that strain E49 only possesses *phaA* (acetyl-CoA acetyltransferase), while the other key genes required for PHA biosynthesis (*phaB* and *phaC*) and decomposition (*phaZ*) were not detected (Fig. S13). Therefore, PHAs are unlikely to function as a carbon or energy storage polymer under the examined conditions.

Collectively, the physiological evidence and genomic features suggest that strain E49 is unlikely to rely on either externally supplied acetate or known autotrophic electron donors to sustain N_2_O reduction under the tested conditions. On the contrary, an enigma remains. The current knowledge of polyphosphate accumulation and degradation (40) concomitantly requires the metabolisms of PHA and glycogen synthesis and degradation. Providing reducing power from the intracellularly accumulated polyphosphate is contingent upon the possession of surrogate pathways for synthesizing and degrading intracellular polymers, which requires identification in the future.

To comprehensively characterize the enzymatic kinetics and enhance the applicability of strain E49 in N_2_O mitigation, future studies should investigate the effects of additional organic substrates beyond acetate on N_2_O reduction activity, identify the endogenous electron donor(s) sustaining respiration under carbon-limited conditions, and perform kinetic assays across a range of initial N_2_O concentrations with extended incubation times to characterize N_2_O affinity and the inhibitory effects of oxygen on nitrous oxide reductase. It should be emphasized that the rates reported in this study represent specific activity under the tested conditions rather than the maximum catalytic potential. Hence, further experimentation is required to establish the maximal N_2_O reduction capacity of strain E49. Furthermore, the duration over which strain E49 can sustain N_2_O reduction in the absence of externally supplied carbon remains unknown. Although the results demonstrate measurable N_2_O reduction activity during the assay period, the intracellular reserves supporting this activity are likely finite. Consequently, prolonged N_2_O reduction would be expected to require replenishment of electron donors. Future studies should therefore investigate the minimum carbon requirements, optimal carbon-loading strategies, and long-term N_2_O reduction performance of strain E49 under environmentally relevant conditions. Such information will be essential for evaluating the practical implementation of strain E49 in sustained N_2_O-mitigation systems.

This study provides the first comprehensive genomic and physiological characterization of *S. frequens* strain E49, an N_2_O-reducing bacterium isolated from activated sludge. The genome encodes both a complete denitrification pathway, including clade I *nosZ*, and a full *ectABCD-ask* cluster for osmoprotectant synthesis, indicating combined respiratory and stress adaptation capabilities. Experimentally, strain E49 demonstrated effective N_2_O reduction under anaerobic conditions without externally supplied organic carbon during the assay period, highlighting its potential role as a biological N_2_O sink. The ability to reduce N_2_O without external organic carbon represents a potential advantage for applications in oligotrophic or engineered systems, such as wastewater treatment. Overall, these findings expand current understanding of *S. frequens* physiology and support further exploration of strain E49 for sustainable N_2_O mitigation and resource recovery strategies.

## MATERIALS AND METHODS

### Enrichment culture using membrane biofilm reactor

To enrich for N_2_O-reducing bacteria, 10 mL of activated sludge obtained from a leachate treatment plant in Tokyo, Japan (41), was inoculated into an MBfR and cultured. The MBfR consisted of a liquid chamber and a gas chamber separated by a flat, gas-permeable silicone membrane (volume 0.26 L; dimensions 170 × 30 mm; thickness 1 mm), as previously described (42). A feed gas containing 5% (vol/vol) N_2_O and 95% (vol/vol) N_2_ was supplied at 5 kPa via the silicone membrane from the gas chamber. Gas flow was regulated via a mass flow controller (Fig. S1). The MBfR was maintained at 30°C in a thermostatic chamber for 604 days.

Modified DSMZ 1180 medium containing methanol and NH_4_Cl as organic carbon and nitrogen sources was continuously supplied and circulated through a side port of the MBfR, as illustrated in Fig. S1, providing nutrients to the liquid chamber where the biofilm develops. The medium was prepared by dissolving 30 g NaCl, 200 mg MgSO_4_·7H_2_O, 20 mg CaCl_2_·2H_2_O, 0.526 g NH_4_Cl, 10 mL CH_3_OH (99.8 wt%), and 1 mL trace element solution in 1 L of distilled water. The trace element solution consisted of 5 g EDTA, 100 mg CuCl_2_·5H_2_O, 2 g FeSO_4_·7H_2_O, 100 mg ZnSO_4_·7H_2_O, 20 mg NiCl_2_·6H_2_O, 200 mg CoCl_2_·6H_2_O, 30 mg Na_2_MoO_4_, 30 mg MnCl_2_·4H_2_O, and 30 mg H_3_BO_3_ per liter. To maintain pH between 8.5 and 9.0, a carbonate buffer (1 M NaHCO_3_, 50 mL/L; 1 M Na_2_CO_3_, 5 mL/L) and a phosphate buffer (KH_2_PO_4_, 14 g; Na_2_HPO_4_·12H_2_O, 30 g per liter) were added at 20 mL/L. Oxygen in the medium was continuously purged with N_2_ in the inlet tank. Hydraulic retention time was maintained at 1 day.

### Isolation by limiting dilution

Pure cultures were isolated by serial dilution in microplate wells containing sterile, modified DSMZ 1180 medium. An aliquot of 20 μL culture was mixed with 180 μL medium per well. Microplates were incubated anaerobically in AnaeroPack (Mitsubishi Gas Chemical Company, Tokyo, Japan) with a headspace gas mixture of 50% CH_4_ and 50% N_2_O (vol/vol). The highest dilution wells showing growth were transferred to fresh microplates, and the procedure was repeated until pure cultures were obtained.

Purity was periodically confirmed by extracting genomic DNA with the FastDNA Spin Kit for Soil (MP Biomedicals, Santa Ana, CA, USA). The V4 region of the 16S rRNA gene was amplified using universal primers (515f and 806r) (43). The Sanger analysis was performed by an analytical service (Fasmac Co., Ltd., Kanagawa, Japan), and the purity was confirmed by visual inspection of the waveform. Finally, the band pattern was analyzed using a polymerase chain reaction restriction fragment length polymorphism to confirm the isolation.

### DNA extraction and genome reconstruction

Isolates were cultivated under batch conditions, and genomic DNA was extracted using the phenol-chloroform method. RNA was removed by RNase A treatment (Takara Bio Inc., Japan). Long-read sequencing libraries were prepared using the 1D ligation sequencing kit (SQK-LSK-109; Oxford Nanopore Technologies Ltd., Oxford, UK) and sequenced on a MinION Mk1B device with R.10 flow cell (FLO-MIN110; Oxford Nanopore Technologies Ltd., Oxford, UK). Basecalling was performed with Guppy v5.0.11 (https://nanoporetech.com/document/Guppy-protocol). Sequence quality was assessed using NanoPlot v1.40.0 (44), and adapter sequences were trimmed using Porechop (https://github.com/rrwick/Porechop). The obtained sequences were assembled using Flye v2.9.2 (45).

Short-read libraries were prepared using the MGI Easy FS PCR Free DNA Library Prep Set (MGI Tech., Shenzhen, China) according to the manufacturer’s protocol. Libraries were generated by a sequencing service (Genome-Lead Co., Ltd., Kagawa, Japan), and 150 bp paired-end sequencing was performed using DNBSEQ-G400FAST (MGI Tech., Shenzhen, China). Adapter sequences, barcode sequences, low-quality reads (less than Q30), terminal 1 base pair, reads less than 20 bp, and reads with *N* ≥ 10 and low-quality ends (3′) were removed using Fastp v0.23.2 (46). The two types of long and short reads were mapped to the assembly obtained with BWA mem, and the SAM file generated with samtools (47) was converted to a BAM file. The genome was obtained by polishing the assembly with Pilon v1.23 (48) using each BAM file.

Genome completeness was assessed using gVolante v2.0.0 (49) with the BUSCO v5 pipeline (50). Clusters of orthologous genes (COGs), coding sequences (CDS), tRNAs, rRNAs, GC content, and GC skew of the obtained genomes were visualized using Genovi v0.4.3 (51).

### Phylogenetic analysis and genome annotation

Phylogenetic classification was performed using GTDB-Tk v2.3.0 (52) and classify_wf (reference data version r214), and a phylogenetic tree of the family *Pseudomonadaceae* was constructed using de_novo_wf with 120 marker genes (outgroup_taxon: --outgroup g__Azomonas, --taxa_filter g__Stutzerimonas). To confirm the novelty of the isolates, the average nucleotide identity (ANI) with related species was calculated using FastANI v1.3.2 (53) included in classify_wf. Genome annotation was performed using DFAST v1.2.17 (54) (--fix_origin --offset 100 --aligner blastp). The obtained amino acid sequences were annotated by mapping them to the KEGG reference metabolic pathways using BlastKOALA v3.1 (55), and functional genes of the isolates were identified. A wide range of functional genes was investigated, with emphasis on gene groups involved in methane, methanol, and N_2_O metabolism during cultivation. Unless otherwise specified, all software used the default parameters.

### Fluorescence microscopy and total cell enumeration

Diluted cell suspensions (1:10) were fixed by immobilization with 25% (vol/vol) glutaraldehyde and subsequently fixed in 99.5% ethanol to preserve cellular integrity. Samples were sonicated in an ultrasonic bath (AS ONE MCU-20, Osaka, Japan) to disperse cell aggregates. A 50-μL aliquot of the treated suspension was vacuum-filtered onto a 0.2-μm pore-size black membrane filter (Isopore, Merck Millipore, Germany) for fluorescence staining.

The morphology and uniformity of strain E49 were examined using SYBR Green I (nucleic acid-binding dye) staining and fluorescence microscopy (BZ-X800, Keyence, Osaka, Japan). Imaging was performed using standard excitation (488 nm) and emission (520 nm) filters.

The filters were stained with SYBR Green I nucleic acid dye (Thermo Fisher Scientific, USA) and mounted on glass slides using SlowFade Diamond Antifade Mountant (Thermo Fisher Scientific). The protocol was adapted from the previous work (34), with minor modifications. Fluorescence imaging was conducted using fluorescence microscopy. Images were acquired at 1,000× total magnification using a high-resolution 100× oil-immersion objective and a 10× digital zoom. To ensure statistical robustness, 40 random microscopic fields were captured per sample, and total cell counts were quantified using BZ-X800 Analyzer software. The cell concentration was calculated using the formula:


(1) $$C\;[cells/mL]=\frac{x}{s\times n}\times S\times \frac{1000}{v}\times m\times \frac{1}{0.91996}\times \frac{1}{0.9}$$

where *x* [–] is the total number of cells enumerated, *s* [mm^2^] is the image area (0.015755 mm^2^) at 1,000× magnification, *n* [–] is the number of micrographs, *S* [mm^2^] is the effective filter area (216.8 mm^2^), calculated from the inner diameter of the filter holder, *v* [µL] is the filtered sample volume, *m* [–] is the dilution ratio, 1/0.91996 is the correction for dilution with glutaraldehyde, and 1/0.9 is the correction for dilution with ethanol

### N_2_O consumption test

A schematic overview of the experimental workflow used for the N_2_O reduction assay is provided in Fig. S6.

### Pre-experimental setup

To assess the influence of cultivation history and medium composition on N_2_O reduction activity, the following three cultivation regimens were evaluated: (i) T1, where both the pre-culture and assay were conducted in DSMZ 1180 medium; (ii) T2, where the pre-culture was conducted in JCM 12 medium and the assay in DSMZ 1180; and (iii) T3, where both steps were performed in JCM 12 medium. Strain E49, stored as a glycerol stock at −80°C, was revived by inoculating 0.1 mL of frozen bacterial stock (0.33% vol/vol) into each pre-culture condition. Following inoculation, all pre-cultures were incubated statically at 30°C until visible turbidity developed (approximately 96 h). Cultures exhibiting an OD_600_ of approximately 0.93 were subsequently used for N_2_O reduction assays. JCM 12 has been commonly used to characterize denitrifying bacteria (34), whereas DSMZ 1180 was originally used for enrichment and isolation.

JCM 12 medium (pH 7.0) contained per liter: 5 g NaCl, 5 g Bacto Peptone, and 3 g Oxoid Lab-Lemco meat extract. DSMZ 1180 medium was prepared as described in “Enrichment culture using membrane biofilm reactor,” above, omitting the carbon source and increasing NH_4_Cl to 1.0 g/L to enable N_2_O consumption without external organic carbon addition during the assay. NH_4_Cl also served as a reduced nitrogen source to support cell growth under nitrate-free conditions, following established strategies for enriching N_2_O-respiring microorganisms (38, 56). For activity assays, DSMZ 1180 medium was modified by replacing KNO_3_ with NH_4_Cl to suppress nitrate-dependent respiration and promote N_2_O as the sole terminal electron acceptor.

#### N_2_O consumption assay

Anaerobic assays were performed in the absence of externally supplied carbon to evaluate the intrinsic N_2_O-reducing capacity of strain E49 independent of organic carbon addition during the assay period. This approach simulates oligotrophic environments, such as advanced activated sludge systems in wastewater treatment plants, where organic carbon availability is often limited and can influence microbial N_2_O reduction (3, 7, 57).

Preliminary tests using a CH_4_/N_2_O (50:50 vol/vol) headspace revealed no detectable CH_4_ consumption (Fig. S4), suggesting the unavailability of strain E49 utilizing CH_4_ as an electron donor. Subsequent gene function analysis confirmed the absence of the *pmoABC* operon (Fig. S3), encoding particulate methane monooxygenase (pMMO), the key enzyme responsible for aerobic methane oxidation. Thus, CH_4_ was excluded from all subsequent N_2_O consumption assays.

Starter cultures (OD_600_ = 0.93) were harvested by centrifugation (10,000 × *g*, 5 min, 4°C) and washed three times with sterile, carbon-limited modified DSMZ 1180 medium to remove residual organic carbon, including glycerol potentially carried over from cryostock preservation. Washed cell pellets were resuspended in 1 mL of fresh DSMZ 1180 medium, and the entire suspension was inoculated into 30 mL of the prepared medium in 120 mL glass vials (Maruemu Corporation, Tokyo, Japan) sealed with anaerobic stoppers (Kanda Rubber Chemicals, Tokyo, Japan) and aluminum crimp caps (GL Sciences, Tokyo, Japan). To estimate the amount of organic carbon potentially introduced through inoculation, an aliquot of the glycerol-based bacterial stock was filtered through a 0.22 μm-pore disposable filter and measured as total organic carbon (TOC) concentration using a TOC-L analyzer (TOC-L CPH, Shimadzu, Kyoto, Japan). This analysis was performed to assess whether carryover organic carbon from the inoculum could have contributed to the observed N_2_O reduction activity.

Anaerobic conditions were established by purging the medium with N_2_ for 10 min prior to sealing. Vials were subjected to five cycles of vacuum degassing (90 s) and helium flushing (30 s) using a vacuum manifold. Pure N_2_O (99.99%) was injected to a final headspace pressure of 170 kPa using a gas displacement apparatus (Model GR-8, Sansin Industrial, Kanagawa, Japan). Medium components susceptible to thermal degradation (NaHCO_3_ and Na_2_CO_3_) and precipitation (phosphate buffer) were filter-sterilized (0.22 µm) and added post-autoclaving to prevent precipitation.

#### Gas analysis

Headspace N_2_O was quantified by gas chromatography-mass spectrometry (GC/MS; GCMS-QP2010 Ultra, Shimadzu Corporation, Kyoto, Japan) equipped with a PoraPLOT Q-HT column (film thickness: 10 μm; length: 25 m; inner diameter: 0.32 mm; Agilent Technologies Japan, Ltd., Tokyo, Japan). Prior to sampling, vial pressures were recorded (PG-100-101GP manometer, Nidec Copal). A 50 µL gas sample was injected via a gas-tight syringe (Hamilton 1700 series) under the following conditions: helium carrier gas (25.3 mL/min), column oven at 50°C, injector at 100°C (2.1 min hold), MS interface at 250°C, and ion source at 200°C.

The N_2_O composition in the headspace was determined from GC/MS peak areas, which were converted to concentrations using calibration curves generated from certified N_2_O standards (R^2^ > 0.999). The partial pressure of N_2_O in the headspace was calculated from the measured internal vial pressure and GC/MS-determined composition. The corresponding dissolved N₂O concentration in the medium was then estimated using the ideal gas law.

All measurements were performed in technical triplicate. The total initial N_2_O amount (headspace + dissolved), calculated using the ideal gas law and measured vial pressures, was 60.0  ±  0.99  µmol across the samples. This total N_2_O value was subsequently used to calculate biomass- and cell-specific N_2_O reduction rates in “Pre-experimental setup,” above.

### Biomass- and cell-specific N_2_O reduction rate estimation

To assess the N_2_O-reducing capacity of *S. frequens* strain E49, the total N_2_O amount (headspace + dissolved) in each vial was measured at multiple time points during a 2.5-h assay period following the establishment of anaerobic conditions and N_2_O addition. The 2.5-h duration was selected to focus on the initial N_2_O consumption kinetics, minimizing the influence of bacterial growth, as preliminary experiments indicated linear N_2_O depletion within this timeframe. The N_2_O consumption rate was calculated at each interval, and the average consumption rate over the experiment was determined as:


(2) $$r_{avg}=\frac{\sum \left({\text{N}}_{2}\text{O consumed in each interval}\right)\;(\mu mol)}{\text{total time}\;(h)}$$

This average rate was then normalized to biomass and cell number as follows.

The dry cell weight (DCW) was determined by harvesting the culture biomass, washing the cells with 0.5× PBS to remove residual medium components, and freeze-drying the biomass using an Eyela FDU-1200 freeze dryer (Tokyo Rikakikai Co., Ltd., Japan) until a constant weight was obtained.

The biomass- (based on DCW) and cell-specific N_2_O reduction rates were calculated using equations 3 and 4, respectively, and expressed in units of [µmol-N_2_O/mg-biomass/h] and [µmol-N_2_O/cell/h]:


(3) $$v_{\text{biomass}}=\;\frac{r_{avg}\text{(}\text{mol-}N_{2}\text{O/h)}}{\text{biomass (mg)}}$$

(4) $$v_{\text{cell}}=\;\frac{r_{avg}\text{(}\text{mol-}N_{2}\text{O/h)}}{\text{cell number}}$$

Total cell counts were quantified using BZ-X800 Analyzer software, and the cell concentration was calculated using equation 1 (see “Fluorescence microscopy and total cell enumeration,” above). These normalized rates represent the specific N_2_O-reducing activity of strain E49 under the tested conditions. Since the experiments were conducted using a single initial N_2_O concentration and did not include a range of substrate concentrations, classical biokinetic modeling (e.g., Michaelis–Menten kinetics) was not applicable. Instead, N_2_O consumption rates were estimated to compare the conditions of T1, T2, and T3.

### Microrespirometric assay

A microrespirometric assay was conducted to evaluate the effect of acetate supplementation, used as a representative organic carbon source, on the respiratory activity of *S. frequens* strain E49 during N_2_O reduction. Cells were first cultivated in JCM No. 12 medium consisting of 5 g/L Bacto Peptone, 5 g/L NaCl, and 3 g/L Lab Lemco meat extract in ultrapure water. Glycerol stock culture was inoculated into the medium at a 1:100 (vol/vol) ratio and incubated at 30°C with shaking at 150 rpm for 34–38 h.

Cells were harvested by centrifugation at 10,000 × *g* for 5 min, washed twice with 0.05× phosphate-buffered saline (PBS), and resuspended in carbon-free RB1 mineral medium. The RB1 medium consisted of KH_2_PO_4_ (100 mg/L), NH_4_Cl (115 mg/L), NaHCO_3_ (118 mg/L), KCl (13.4 mg/L), MgSO_4_·7H_2_O (8.2 mg/L), NaCl (6.6 mg/L), and 1 mL/L trace element solution. The trace element solution contained FeSO_4_·7H_2_O (10 g/L), FeCl_3_·7H_2_O (10 g/L), ZnSO_4_·7H_2_O (2 g/L), CuSO_4_·7H_2_O (4 g/L), Na_2_MoO_4_·2H_2_O (0.5 g/L), MnCl_2_·4H_2_O (0.1 g/L), H_3_BO_4_ (0.1 g/L), Na_2_SeO_3_ (0.3 g/L), and citric acid (10 g/L). The initial cell density was adjusted to an OD_600_ of 0.26.

The cell suspension was transferred into a 10-mL double-port glass chamber (MR-Ch double port) equipped with a magnetic stirrer and maintained at 30°C using a temperature-controlled water bath, following the microrespirometric setup as previously described (34). Dissolved O_2_ and N_2_O concentrations were continuously monitored using a microrespirometry system equipped with Clark-type amperometric N_2_O (N_2_O-MR) and O_2_ (OX-MR) microsensors (Unisense, Aarhus, Denmark). Sensor signals were continuously recorded using SensorTrace Suite version 3.4.300.19348 (Unisense, Aarhus, Denmark). Prior to measurement, the suspension was stirred at 600 rpm for 5 min to ensure homogeneous conditions. Subsequently, 25–30 μL of N_2_O-saturated water was injected to achieve an initial N_2_O concentration of approximately 60 μM. After 5 min, the stirring speed was reduced to 300 rpm for the remainder of the assay. Whenever reagents were added during the experiment, the stirring speed was temporarily increased to 600 rpm for 5 min before returning to 300 rpm.

Following the depletion of dissolved oxygen, 25 μL of sodium acetate solution was added to achieve a final acetate concentration of 625 μM. After the initial N_2_O was nearly depleted, 50 μL of JCM No. 12 medium was added as a positive control to verify that the cells remained metabolically active. A second aliquot of N_2_O-saturated water was subsequently injected to evaluate respiratory activity following JCM No. 12 supplementation. Dissolved O_2_ and N_2_O concentrations were continuously monitored throughout the experiment.

## Data Availability

The complete genome sequence of *S. frequens* strain E49 has been deposited in NCBI/ENA/DDBJ under BioProject accession number PRJDB35216 and BioSample accession number SAMD00920862. Raw sequencing reads are available under accession numbers DRX671565 and DRX671566, with corresponding run accession numbers DRR691463 and DRR691464.

## SUPPLEMENTAL MATERIAL

Supplemental material aem.00743-26-s0001.docx Fig. S1 to S13; Table S1.

ASM does not own the copyrights to Supplemental Material that may be linked to, or accessed through, an article. The authors have granted ASM a non-exclusive, world-wide license to publish the Supplemental Material files. Please contact the corresponding author directly for reuse.
