# Supplementary material for: Genomic and physiological characterization of newly isolated nitrous oxide-reducing bacterium Stutzerimonas frequens strain E49 from landfill leachate-treating activated sludge
Source: Appl Environ Microbiol. 2026 Aug 31;92(9):e00743-26. doi: 10.1128/aem.00743-26 (PMC13599507; doi:10.1128/aem.00743-26)
Supplement: Supplementary file 1 [file aem.00743-26-s0001.docx]

**Supplementary Information**

**Genomic and Physiological Characterization of a Newly Isolated Nitrous Oxide-Reducing Bacterium *Stutzerimonas frequens* Strain E49 from Landfill Leachate-Treating Activated Sludge**

Jameil Magomnang Jr.,^a^* Shohei Yasuda,^b,c^# Akihiko Terada^a,c^#

^a^Department of Applied Physics and Chemical Engineering, Tokyo University of Agriculture and Technology, Koganei, Tokyo, Japan

^b^Department of Civil Engineering, University of Galway, University Road, Galway, Ireland

^c^Global Innovation Research Institute, Tokyo University of Agriculture and Technology, Fuchu, Tokyo, Japan

Running Head: N_2_O Reduction by *Stutzerimonas frequens* strain E49

#Address correspondence to:

- Akihiko Terada, [akte@cc.tuat.ac.jp](mailto:akte@cc.tuat.ac.jp)
- Shohei Yasuda, [shohei.yasuda@universityofgalway.ie](mailto:shohei.yasuda@universityofgalway.ie)

Present address: Jameil Magomnang Jr., Department of Civil and Environmental Engineering, Nagaoka University of Technology, Nagaoka, Niigata, Japan

**Fig. S1** Schematic diagram of the membrane biofilm reactor (MBfR) used for enrichment of N_2_O-reducing bacteria. The reactor consisted of a liquid chamber and a gas chamber separated by a flat-sheet gas-permeable silicon membrane (volume 0.26 L; dimensions 170 × 30 mm; thickness 1 mm). A feed gas mixture containing 5% (v/v) N_2_O balanced with N_2_ was supplied at 5 kPa through the gas chamber, with flow regulated by a mass flow controller. The MBfR was operated at 30°C in a thermostatic chamber for 604 days.

**Fig. S2** KEGG pathway mapping of amino acid sequences from *Stutzerimonas frequens* strain E49. The genome was annotated and aligned against the KEGG reference pathway for denitrification. Strain E49 possesses the complete set of key denitrification genes, encoding nitrate reductase (*narGHI*), nitrite reductase (*nirS*), nitric oxide reductase (*norBC*), and nitrous oxide reductase (*nosZ*), indicating its complete denitrification potential.

**Fig. S3** KEGG pathway mapping of the methane oxidation pathway reveals the absence of key genes in *Stutzerimonas* *frequens* E49. Essential genes encoding particulate (*pmoABC*) and soluble (*mmoXYBZC*) methane monooxygenases, as well as downstream methanol (*mxaFI*) and formaldehyde dehydrogenases, are not detected in the genome. This confirms that *S. frequens* lacks the genetic capacity for methane oxidation and does not function as a methanotroph.

**Fig. S4** Temporal profiles of dissolved methane (CH_4_) and nitrous oxide (N_2_O) concentrations in control vials containing sterile medium and sample vials inoculated with *Stutzerimonas* *frequens* strain E49 cells. The decline in dissolved CH_4_ concentrations observed in both control and sample vials is attributed to abiotic factors such as gas dissolution and sampling-related losses. No significant biological methane oxidation by strain E49 was detected. Dissolved N_2_O concentrations are shown to illustrate biological N_2_O reduction activity by strain E49. Sample dissolved CH_4_ concentrations were corrected using control data to account for non-biological losses.

Dissolved CH_4_ data are presented with solid blue lines and square markers (■) for controls, and solid orange lines and circle markers (●) for samples. Dissolved N_2_O data are depicted with solid purple lines and square markers (■) for controls and solid red lines with triangle markers (▲) for samples. Dissolved CH_4_ concentrations are plotted on the left y-axis (mg-CH_4_/L), and dissolved N_2_O concentrations on the right y-axis (mg-N_2_O/L). Error bars indicate standard deviations of triplicate measurements (n=3).

**Fig. S5** KEGG pathway mapping of *Stutzerimonas frequens* strain E49 genome shows the presence of the *ectABCDask* gene cluster responsible for the biosynthesis of compatible solutes ectoine and hydroxyectoine, which act as osmoprotectants. The strain lacks the *doeAB* genes associated with ectoine biodegradation, suggesting the dedicated ability to accumulate these osmolytes.

**Fig. S6** Schematic overview of the vial experimental workflow used for evaluating N_2_O reduction by *Stutzerimonas frequens* strain E49. Frozen glycerol stocks were revived under three cultivation regimes (T1–T3), followed by pre-cultivation, cell harvesting and washing, anaerobic assay preparation, N_2_O addition, and N_2_O reduction measurements. N_2_O reduction rates were calculated from measurements obtained during the initial 2.5-h assay period.

**Fig. S7** KEGG pathway mapping of pyruvate metabolism in Stutzerimonas frequens strain E49 highlighting the acetate activation pathway. Genome annotation identified genes encoding acetate kinase (EC 2.7.2.1; ***ackA***), phosphate acetyltransferase (EC 2.3.1.8; ***pta***), and acetyl-CoA synthetase (EC 6.2.1.1; ***acs***), indicating the genetic potential to convert acetate into acetyl-CoA via both the AckA–Pta and Acs pathways. Yellow stars denote acetate and acetyl-CoA, illustrating the metabolic entry point of acetate into central carbon metabolism. **Acetyl-CoA subsequently enters the citrate cycle (KEGG map00020).**

**Fig. S8** **Microrespirometric profile of** Azospira **sp. I13 during N_2_O reduction with acetate supplementation.** Dissolved oxygen (O_2_, blue line) and N_2_O (orange line) concentrations were continuously monitored over time. Acetate was added during the assay to evaluate the respiratory response to an external organic carbon source (indicated by a red arrow). This experiment served as a positive control for comparison with Stutzerimonas frequens strain E49, demonstrating that the microrespirometric assay was capable of detecting respiratory responses following acetate supplementation.

**Fig. S9** KEGG pathway mapping of Stutzerimonas frequens strain E49 genome shows the presence of the **cys gene cluster** responsible for assimilatory sulfate reduction, enabling biosynthesis of sulfur-containing amino acids. The strain lacks the complete oxidative sulfur pathway, indicating that it cannot perform chemolithotrophic sulfur oxidation for energy generation.

**Fig. S10** KEGG pathway mapping of Stutzerimonas frequens strain E49 genome shows the presence of genes involved in oxidative phosphorylation, including polyphosphate kinase 1 (EC 2.7.4.1, PPK1), which catalyzes the reversible conversion between ATP and polyphosphate. This enzyme may contribute to intracellular energy storage and ATP regeneration, supporting metabolic activity under carbon-limited conditions.

**Fig. S11** KEGG pathway mapping of Stutzerimonas frequens strain E49 genome shows the presence of exopolyphosphatase (EC 3.6.1.11, PPX) within the purine metabolism pathway, responsible for hydrolyzing polyphosphate into inorganic phosphate. Together with PPK1, PPX suggests the operation of a polyphosphate cycle, potentially enabling the mobilization of stored energy to sustain processes such as N_2_O reduction in the absence of external carbon sources.

**Fig. S12** KEGG pathway mapping of Stutzerimonas frequens strain E49 genome shows the presence of glycogen synthase (EC 2.4.1.21) and branching enzyme (EC 2.4.1.18), indicating partial capability for glycogen elongation and branching. The enzyme ADP-glucose pyrophosphorylase (EC 2.7.7.27), which synthesizes the glycogen precursor ADP-glucose, is absent and thus **not highlighted in green**, suggesting that complete glycogen biosynthesis is not possible in this strain. However, the glycogen degradation gene glycogen phosphorylase (EC 2.4.1.1) is present.

**Fig. S13** KEGG pathway mapping of the *Stutzerimonas frequens* strain E49 genome shows the presence of phaA, encoding acetyl-CoA acetyltransferase (EC 2.3.1.9), which catalyzes the first step of PHA biosynthesis. However, the key genes phaB and phaC, encoding acetoacetyl-CoA reductase (EC 1.1.1.36) and PHA synthase (EC 2.3.1.304), respectively, were not detected and are therefore not highlighted in green. In addition, the PHA depolymerase gene phaZ (EC 3.1.1.75) was absent. These results indicate that strain E49 lacks a complete PHA biosynthesis and degradation pathway, suggesting that PHAs are unlikely to play a major role as intracellular carbon or energy storage polymers under the conditions examined in this study.

**Table S1**

Total organic carbon (TOC) concentrations of assay medium at different dilution factors.

| Dilution Factor | Replicate 1 | Replicate 2 | Mean ± SD |
| --- | --- | --- | --- |
|  | [mg/L] | [mg/L] | [mg/L] |
| 20× | 15.75 | 15.49 | 15.62 ± 0.18 |
| 100× | 3.187 | 3.195 | 3.191 ± 0.01 |
| 200× | 1.734 | 1.618 | 1.676 ± 0.08 |

Total organic carbon (TOC) concentrations measured in the assay medium at multiple dilution factors (n=2). Based on dilution-corrected concentrations and the estimated effective cultivation dilution (~1:27,000) resulting from three washing steps prior to inoculation, the residual TOC concentration under assay conditions was approximately 0.012 mg/L.
